# Discrimination learning with variable stimulus 'salience'

**DOI:** 10.1186/1755-7682-4-26

**Published:** 2011-08-03

**Authors:** Mario Treviño, Efrén Aguilar-Garnica, Patrick Jendritza, Shi-Bin Li, Tatiana Oviedo, Georg Köhr, Rodrigo J De Marco

**Affiliations:** 1 Department of Molecular Neurobiology, Max Planck Institute for Medical Research, Jahnstrasse 29, 69120, Heidelberg, Germany; 2 Developmental Genetics of Nervous System, Max Planck Institute for Medical Research, Jahnstrasse 29, 69120, Heidelberg, Germany; 3 Departamento de Química, Universidad Autónoma de Guadalajara, 1201 Av. Patria, 44100 Guadalajara, Jalisco, México

**Keywords:** Associative learning, discrimination, salience, associability, behavior

## Abstract

**Background:**

In nature, sensory stimuli are organized in heterogeneous combinations. Salient items from these combinations 'stand-out' from their surroundings and determine what and how we learn. Yet, the relationship between varying stimulus salience and discrimination learning remains unclear.

**Presentation of the hypothesis:**

A rigorous formulation of the problem of discrimination learning should account for varying salience effects. We hypothesize that structural variations in the environment where the conditioned stimulus (CS) is embedded will be a significant determinant of learning rate and retention level.

**Testing the hypothesis:**

Using numerical simulations, we show how a modified version of the Rescorla-Wagner model, an influential theory of associative learning, predicts relevant interactions between varying salience and discrimination learning.

**Implications of the hypothesis:**

If supported by empirical data, our model will help to interpret critical experiments addressing the relations between attention, discrimination and learning.

## Background

In nature, sensory stimuli are organized in heterogeneous combinations. Salient items from these combinations 'stand-out' from their surroundings and influence what and how we learn. The salience of these items arises from the joint action of the items' intrinsic physical properties and the motivational state of the subject that learns about them; ultimately, it determines the discriminative-incentive value of such items [1-3]. In psychophysics, perceptual thresholds of detection and discrimination are estimated by means of linear variations of stimulus properties from a level of 'no detection', to a level of 'robust detection', and vice versa [4]. The sign and slope of these variations are not expected to interfere with the decoding capabilities that serve the setting of perceptual detection [5]. Yet, stimulus salience is subject to variation as learning occurs, and multiple items compete for attention. From the point of view of discrimination learning, the relationship between varying salience and learning remains unclear.

For the past four decades, the Rescorla-Wagner (RW) model [6] has been a very influential theory of associative learning. It explains how the associative status of a conditioned stimulus (CS) varies when it is trained, i.e., repeatedly paired with an unconditioned stimulus (US) [6,7]. Equation 1 shows the model as proposed by the authors:(1)

Where *V*(*t*) is the strength of the CS-US association or the cumulative amount of learning,  is the CS salience (0 ≤ *α *≤ 1), *β *corresponds to US salience (0 ≤ *β *≤ 1) and *λ *is the asymptote of learning, i.e., maximum retention level at infinite training repetitions. This model predicts that the development of a conditioned response will depend upon sustained changes in the strength of the CS-US association. In each learning trial, the change in *V*(*t*) will be proportional to the product between *α*, *β *and the difference between λ (set by specific attributes of the US) and the sum of *V*(*t*) for all the stimuli present in the trial. Thus, the strength of the CS-US association and the degree of learning towards the CS will increase throughout successive learning trials in a negatively accelerated fashion, as *V*(*t*) approaches *λ*.

The RW-model has been influential because it is simple and allows predictions in situations where multiple cues are reinforced simultaneously, accounting for learning phenomenah as blocking and overshadowing [7]. Yet, while the RW-model assumes a constant processing of CS information, in nature, CS (and US) salience is subject to variation. Indeed, there is general agreement that the salience of any given CS (or conditioned situation) will depend on: (i) the physical properties of the environment that determine how discriminativeis the CS (as it stands against a background), as well as on (ii) subject- and motivation-dependent perceptual features that influence learning [8,9]. In other words, *α *depends on constellations of sensory inputs and the subject's information capabilities, but it also varies with experience and motivation. Ultimately, the joint action of these external and internal elements will determine whether and how the CS is assigned with a particular predictive value.

## Presentation of the hypothesis

In the laboratory, learning is easier to predict when training stimuli and motivational states are kept as constant as possible, a most unlikely situation in real life. In nature, open environments vary and afford locomotion, changing the structure of sensory arrays [10]. Salience is strongly influenced by the interplay between locomotion, perception, past experience and acquired knowledge. Irrespective of the physical properties of the stimulus in question, CS associability is not immutable because reinforcement modifies incentive values and leads to complex interactions between sensory inputs and conditioned responses [1]. Thus, a rigorous formulation of the problem of discrimination learning should account for varying CS salience and perceptibility. We hypothesize that controlled variations of the environment will modify CS salience and determine learning rates and retention values in a predictable manner. As the subject learns at different rates, this may lead to different computational strategies to discriminate objects from the sensory stream. We subscribe to the idea that theoretical models of learning can guide experimental design. We here explore the validity of our hypothesis by means of a modified version of the RW-model accounting for varying CS salience effects.

## Testing the hypothesis

Let us modify the RW-model to account for varying CS salience, as well as to include a putative discrimination threshold in the following equation:(2)(3)

Where *α*(*t*) represents variable salience over time and *α*_min _is the salience threshold for learning to occur. For simplicity, we represent *λ *as a sliding logistic function of *α *[11], because the quality of sensory representation should degrade gradually as salience reaches *α*_min_, compromising discrimination [12] and learning. We assume that discrimination performance is constrained by a perceptual grid that filters out relevant information for the discrimination task, as represented by *λ*(*α*) at low *α *values.

However, λ could also be modeled using a Boltzmann distribution [5], or other functions [13,14]. (Note that additional variants on the model have been addressed elsewhere [7,15]).

**Figure 1:**
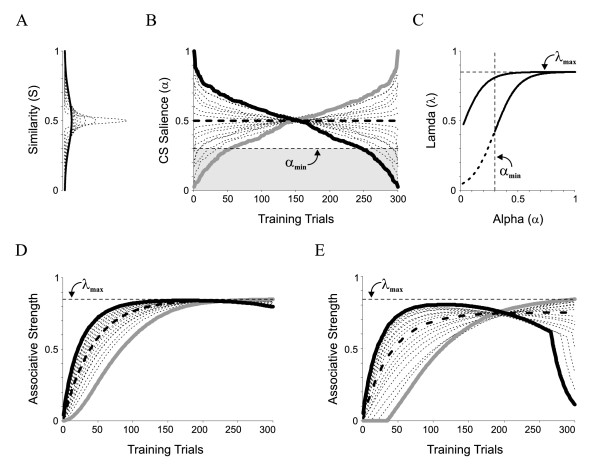
**Learning with varying CS salience**. (A) We generated stimuli with variable degrees of similarity using random numbers from a set of normal distributions with **fixed mean **(μ = 0.5) and variable standard deviations from 0 to 0.18, with 0.02 steps (σ = 0:0.02:0.18). (B) To simulate discriminative training, stimuli were sorted according to either increasing (gray) or decreasing (black) salience (Note that such arrangements consist of the same stimuli). The shaded region covers salience levels below an arbitrary putative threshold for learning of α_min _= 0.3. (C) The asymptote of learning, λ, as presented in Eq. 3, behaves as a constant (λ ≈ λ_max_) for highly salient items, but drops and becomes sensitive to gradients in α as α reaches α_min_. We used two salience threshold levels, namely, α_min _= 0 and 0.3, which led to the left and right sigmoid curves, respectively. (D-E) Predicted learning curves for stimuli with increasing (gray) or decreasing (black) salience as arranged in (B), with α_min _= 0 (D), and α_min _= 0.3 (E). The differences in the learning curves (black vs. gray) are due to the arrangement of varying salience used during training. **Learning curves were identical to those predicted by the standard model when similarity was held constant (thick dotted lines)**. Discrete, numerical solution to the equations is displayed as continuous lines for visualization purposes.

Regarding varying salience: if stimulus 'i' is reinforced, then *α_i_*(*t*) should increase, and if stimulus 'j' is not reinforced, then *α_j_*(*t*) should decrease. In a situation where the stimuli, 'i', 'j', and 'k' are sequentially reinforced, then an increase in a *α_i_*(*t*) should affect *α_j_*(*t*) and *α_k_*(*t*) according to the degree of similarity between the stimuli. Therefore, the varying salience over time may adopt the following form:(4)

where S_i,j _represents the degree of similarity between the i^th ^(reference) and j^th ^stimuli (0 ≤ S ≤ 1), and *α_i_*(*t*) is the dynamic representation of salience with respect to item 'i', as the probability of attention will vary together with salience and learning [16,17]. Thus, *α *(*t*) should increase or decrease depending on both, reinforcement levels and the temporal arrangement of stimuli similarity during training. Evidently, we do not know how salience evolves with learning. Let us consider a simple steady-state scenario, where *α *(*t*) equals S_i,j_. What would be the effect of varying stimuli similarity during learning? To explore this idea, we first generated a set ofstimuli with different degrees of similarity by using random numbers from normal distributions with fixed meanand variable standard deviations (Figure 1A). These numbers represent training stimuli with different salience. To investigate whether variable salience has a relevant effect in learning, we sorted the stimuli using other decreasing (black line) or increasing (gray line) similarity (Figure 1B). These arrangements maximize the relative difference in salience between training programs but consist of exactly the same stimuli. Next, we calculated *λ*(*α*), applying either no salience threshold (i.e. *α*_min _= 0) or a putative threshold of 0.3 (*α*_min _= 0.3; Figure 1C). Panels D-E show the predicted learning curves, as given by Eq.2. In all cases of identical mean salience of 0.5, the temporal arrangement of training stimuli determined the shape of the learning curves.

Moreover, when discriminative training involved stimuli below the salience threshold for learning (Figure 1E), stimuli with salience below *α*_min _were undetectable, *V*(*t*) did not increase (for *V*(*t*) = 0), and the curves decayed in a mono-exponential manner due to the lack of reinforcement (0 ≤ *V*(*t*) ≤ 1). When similarity was held constant (thick dotted lines), the learning curves were identical to those predicted by the standard model.

## Implications of the hypothesis

In order to survive, organisms must learn to discriminate items with predictive values. Some models of associative learning assume a processing of conditioned stimuli with constant salience [6], but in nature salience is variable as environments and experience change dynamically. Some theories emphasize that multiple CSs must compete for internal representations of limited capacity, forcing learning about some stimuli to be at the expense of learning about other stimuli [1]. A realistic formulation of the problem of learning must consider varying CS salience, not only because learning exerts a direct influence on it (via attention and contiguity), but also because discriminative stimuli exchange and compete for attention. Using numerical simulations of discriminative training, we here show that a modified version of the Rescorla-Wagner model predicts how varying CS salience influences discrimination learning. This interaction may become evident in conditions where discrimination learning is slow and multiple arrangements of training stimuli are compared, as we did here. If true, such a mathematical variant may become useful to explain the co-varying interactions between attention, discrimination and learning. A general learning theory must address the internal and external factors that influence how the brain allocates attention and apprehends the environment to select, store and retrieve information for generating adaptive behavior.

## Competing interests

The authors have no financial competing interests.

## Authors' contributions

MT and RM: conceived ideas. MT and EA: developed the equations and simulations in MATLAB 7.8 (MathWorks, Inc.; Natick, USA). All authors contributed writing and revising the manuscript. All authors read and approved the final version of the manuscript.
